# Necrotizing soft tissue infection of the scalp and face

**DOI:** 10.1080/23320885.2025.2575330

**Published:** 2025-10-16

**Authors:** Sakar Gupta, Pradeep K. Attaluri, Jeffrey Larson, Ahmed M. Afifi

**Affiliations:** Division of Plastic and Reconstructive Surgery, University of Wisconsin School of Medicine and Public Health, Madison, WI, USA

**Keywords:** NSTI, scalp, face

## Abstract

Introduction: Necrotizing soft tissue infections (NSTIs) are characterized by rapidly spreading, life-threatening infections with widespread soft tissue necrosis that most commonly infect the extremities, torso, and perineum. Although rare, NSTIs of the head and neck are particularly dangerous given the complex anatomy surrounding the region and demand early diagnosis, antibiotic administration, and surgical debridement. Case presentation: We report a case of a 64-year-old male who presented with a NSTI of the scalp and face after sustaining a laceration to the posterior scalp. The patient initially presented at his local emergency department with subtle and nonspecific clinical signs and unrevealing imaging, which represented a diagnostic challenge for early intervention. Upon presentation to our facility’s emergency department three days later, the patient had already started to develop severe sepsis and systemic involvement. As a result, despite intensive supportive care and surgical debridement, the patient’s clinical course was complicated by multisystem organ failure and death. Discussion: Our case highlights the importance of maintaining a high clinical index of suspicion for NSTIs in patients with soft tissue infections of the head and neck, even when hallmark features are absent. Timeliness to intervention remains the single most important factor in determining survival.

## Introduction

Necrotizing soft tissue infections (NSTIs) are aggressive, life-threatening soft tissue infections that rapidly spread across fascial planes [1–9]. They are characterized by widespread necrosis that can extend from the dermis to the deep muscle. NSTIs are often polymicrobial in nature, involving synergistic activity of anaerobic and aerobic microorganisms, and are often associated with risk factors such as traumatic wounds, congestive heart failure, immunosuppression, or diabetes [1,6,8]. Although NSTIs most commonly affect the extremities, perineum, and torso, cases involving the head and neck are particularly dangerous due to the region’s complex anatomy and proximity to the airway, cranial nerves, and central nervous system [5]. If not recognized and treated promptly, these infections can rapidly progress to systemic toxicity and septic shock, contributing to high rates of morbidity and mortality [1,4,6,10].

Early management is crucial to improving patient outcomes related to NSTIs, particularly for the NSTIs of the head and neck, which are rarer compared to the more common presentations in the trunk and extremities [1,3,6,10]. However, the systemic manifestations of sepsis and multiorgan failure are usually preceded by early subtle and nonspecific clinical signs – erythema, swelling, and severe pain – which can impede timely diagnosis [1–4,6,11]. Current guidelines emphasize prompt recognition and intervention, including intensive supportive care, aggressive surgical debridement of the infected and necrotic tissue, and broad-spectrum antibiotics [1–4,6,10]. However, despite advancements, mortality rates of NSTIs range from 20% to upwards of 76% in settings of delayed intervention and complications with sepsis and mediastinitis [7,9,11,12].

We present a case of a 64-year-old male with a severe NSTI of the head and neck whose clinical course was complicated by multi-system organ failure and death.

## Case presentation

**Figure 1.**
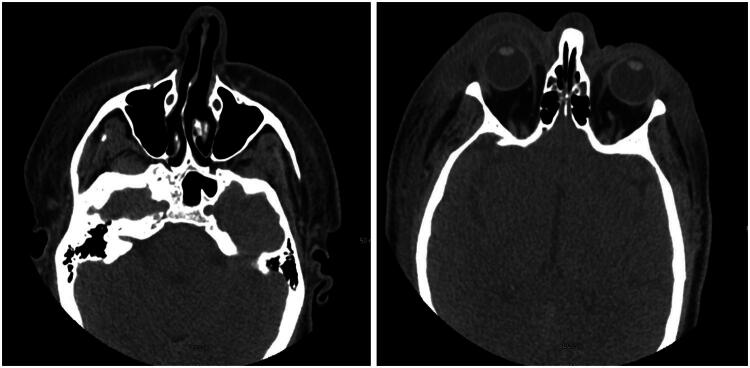
CT maxillofacial scan with findings concerning for a subcutaneous soft tissue infection.

A 64-year-old male with a past medical history of congestive heart failure (LVEF 25-30%), hypertension, paroxysmal atrial fibrillation (s/p DCCV on Eliquis), and non-ischemic cardiomyopathy was transferred to our institution’s emergency department (ED) due to concerns of an NSTI of the scalp and face. Eight days prior to presentation at our tertiary care facility, the patient sustained a laceration to the posterior scalp while operating a skid loader. He did not seek medical attention at that time. Five days after this injury, the patient presented to his local ED for continued pain secondary to his scalp laceration. A non-contrast CT head was performed, which was normal, and the patient was subsequently discharged without antibiotics after having the laceration closed on a delayed basis. Three days later, now 8 days after the initial injury, the patient returned to the ER, reporting increased swelling, erythema, and a new onset of altered mental status. A repeat CT maxillofacial scan (Figure 1) now demonstrated significant soft tissue stranding and gas in the scalp and facial soft tissues. Within 2 h of presentation, the patient began to develop hemodynamic instability and went into atrial fibrillation with RVR and was treated with diltiazem and Zosyn for presumed infection. He was promptly transferred to our institution’s ED for further evaluation and management.

**Figure 2.**
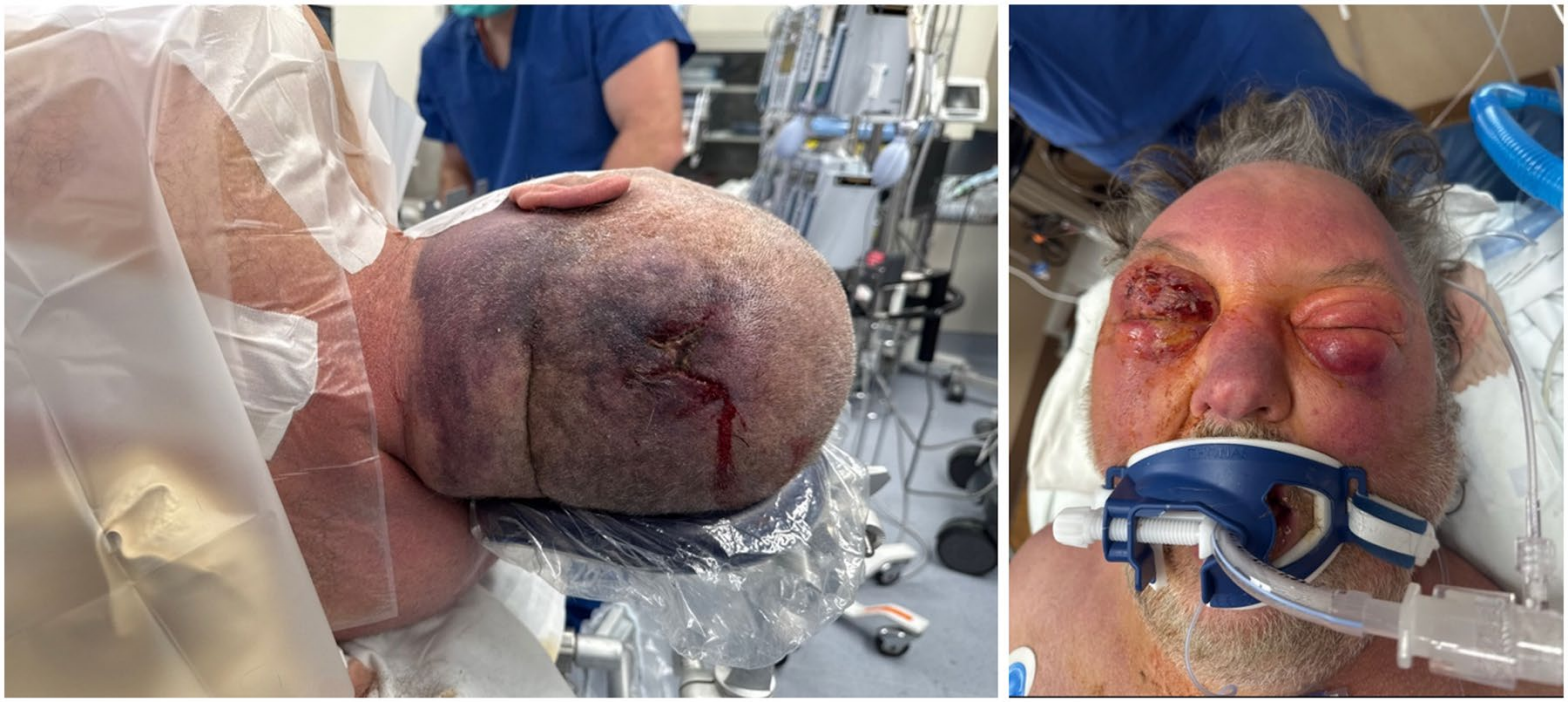
Clinical photographs at presentation demonstrating marked facial swelling with periorbital involvement.

On arrival at our ED, the patient’s mental status continued to deteriorate, and he was emergently intubated for airway protection. He was febrile, tachycardic in atrial fibrillation with rapid ventricular response, and was unable to open his eyes bilaterally due to significant facial edema (Figure 2). Laboratory evaluation (Table 1) revealed evidence of lactic acidosis, acute kidney injury, transaminitis, hyperbilirubinemia, coagulopathy, and hypoglycemia – concerning for sepsis. Linezolid was also administered for presumed necrotizing soft tissue infection. The patient was emergently taken to the operating room by multiple teams (ENT, Plastic Surgery, Ophthalmology, Surgical Intensive Care Unit, and Emergency General Surgery) for extensive soft tissue debridement within 38 min of arrival at the ED.

**Table 1.** Initial laboratory findings upon arrival to our facility’s ED.

Laboratory values	
Lactate	8.6 mmol/L
BUN	72 mg/dL
Creatinine	2.51 mg/dL
Sodium	131 mmol/L
Calcium	8.0 mg/dL
C-reactive protein	40.2 mg/dL
Procalcitonin	18.15 ng/ml
Glucose	55 mg/dL
AST	429 U/L
ALT	369 U/L
Total bilirubin	7.2 mg/dL
INR	2.7

**Figure 3.**
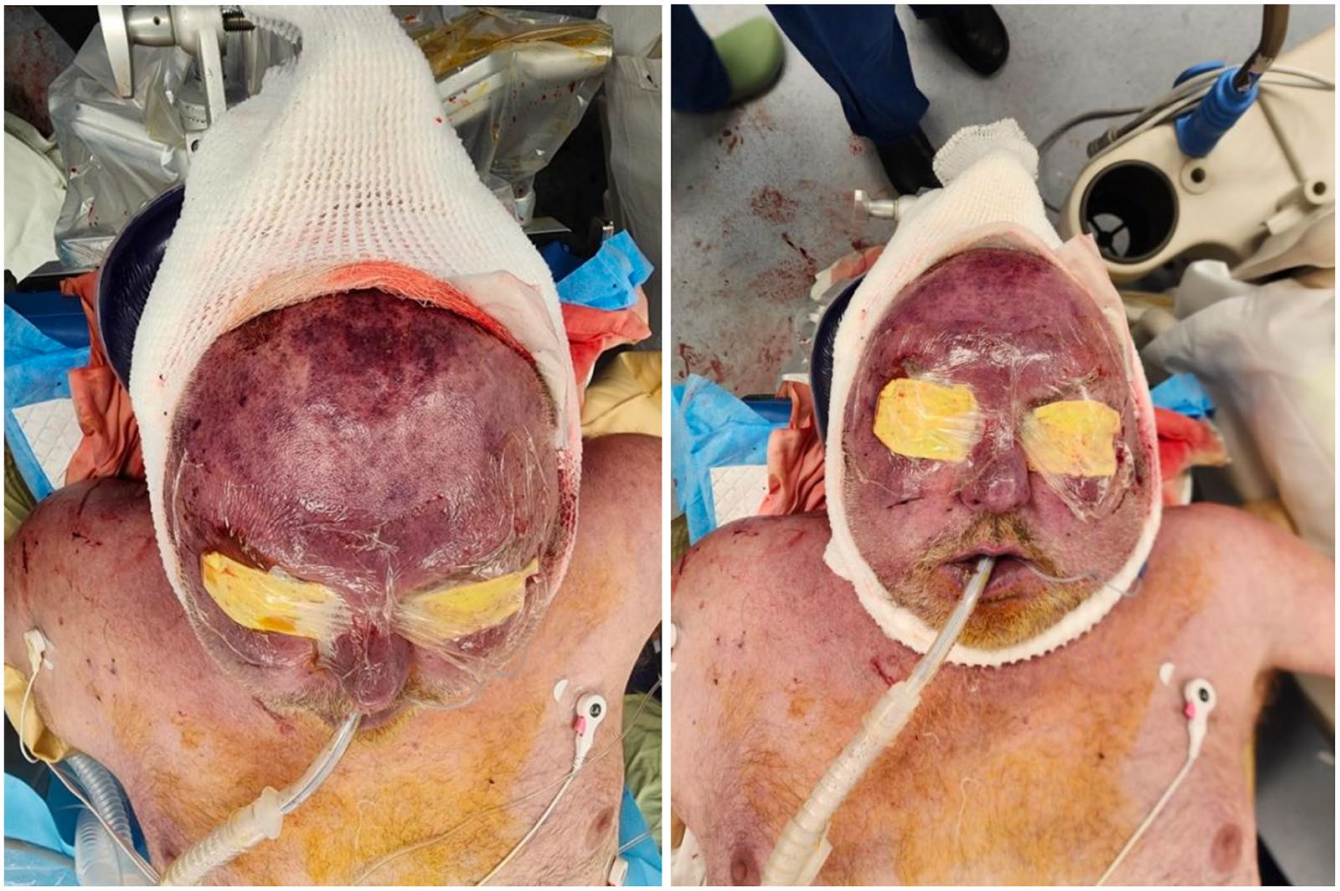
Postoperative photographs following emergent debridement.

The debridement began in the posterior scalp at the location of the initial injury. Dishwasher drainage was immediately encountered. Tangential excision continued anteriorly in a subgaleal plane until the hairline was reached. At this point, the oculoplastic team discovered purulence in the patient’s bilateral upper and lower eyelids with extension superiorly towards the hairline. Given the rapid progression to the anterior face and the patient’s severe hemodynamic instability, the decision was made with anesthesia to terminate the procedure and admit to the Intensive Care Unit (ICU) (Figure 3).

On arrival to the ICU, the patient continued to deteriorate into multisystem organ failure, severe lactic acidosis, liver dysfunction (coagulopathy and hypoglycemia), respiratory failure, and renal failure. Despite aggressive surgical and medical efforts, the patient’s status continued to deteriorate and, in accordance with the wishes of the family, the decision was made to transition to comfort care. The patient expired the following morning.

## Discussion

NSTIs are severe, life-threatening infections of soft tissues leading to extensive necrosis and systemic involvement [1–12]. NSTIs are considered medical and surgical emergencies; however, the initial diagnosis is often delayed because of nonspecific and variable clinical manifestations [12]. Without timely and accurate diagnosis and early medical and surgical intervention, NSTIs are associated with a mortality rate of upwards of 76% [12]. Furthermore, NSTIs of the head and neck pose unique challenges due to the potential involvement of critical anatomical structures that facilitate fascial spread. The likelihood of these infections to progress to severe sepsis is increased with the presence of comorbidities such as diabetes, immunosuppression, alcohol use, congestive heart failure, and chronic kidney disease [12]. One particular concern are the valveless emissary veins that connect the extracranial and the intracranial spaces. Infections that originate in more superficial regions of the head and neck can travel and spread to deeper intracranial structures through these veins, further compromising clinical prognosis [13]. The case described highlights the poor prognosis of NSTIs of the scalp and face and emphasizes the need for a high index of suspicion and subsequent immediate intervention.

The three cornerstones of successful management of NSTIs include early diagnosis, broad-spectrum antibiotics, and emergent surgical debridement [12]. NSTIs initially present as a nonspecific triad of swelling, severe pain, and erythema, which makes early recognition challenging [12]. However, a notable finding is severe pain out of proportion to clinical findings [12]. If presenting late, the patient's condition is often complicated by septic shock, soft-tissue necrosis, altered mental status, and multiorgan dysfunction [12]. NSTIs of the scalp and face, in particular, may also present with peau d’orange skin, blistering and bullae, rapidly developing soft tissue crepitus, and variable skin changes, ranging from cyanotic to overtly necrotic [3,5,14]. In this patient, initial symptoms of localized pain, swelling, and erythema were extremely subtle and nonspecific. Despite progressive deep tissue necrosis, the overlying skin appeared relatively benign. There was no purulence or dishwater-like drainage, and there was no bruising, skin necrosis, crepitus, or foul odor. The lack of these typical features further exaggerated the inherent diagnostic challenge of early recognition of an NSTI in the head and neck. By the time the patient presented to our facility’s ED, he had already started to develop severe sepsis and multiorgan failure, which severely limited the impact of early medical and surgical management.

Laboratory findings may also be helpful in the diagnosis of NSTIs of the face and scalp. Leukocytosis (white blood cells > 20,0000/L), elevated renal labs (BUN > 18 mg/dL and Cr > 1.2 mg/dL), and elevated creatinine kinase (> 16 mg/dL) and C-reactive protein (> 600 IU/L) raise strong suspicion for NSTIs. The Laboratory Risk Indicator for Necrotizing Fasciitis (LRINEC) is a scoring tool that uses routine labs to help classify patients into risk categories for NSTIs [12,15]. However, in patients with multiple comorbidities, including heart failure, immunosuppression, and chronic kidney disease, the diagnostic value of the LRINEC score decreases due to a lack of a robust inflammatory response [12]. Current guidelines recommend immediately proceeding to operative debridement if clinical history and physical exam raise high suspicion for NSTI regardless of the LRINEC score [15]. In this case, the calculated LRINEC score was 8 points, suggesting a high risk for NSTI with a positive predictive value of 93.4% [15]. Furthermore, laboratory findings of lactic acidosis, coagulopathy, and oliguric acute kidney injury (stage 3) were consistent with advanced sepsis and prompted immediate surgical intervention.

NSTIs affecting the extremities, trunk, and perineum are often polymicrobial in nature and mostly impact immunocompromised patients [10,12]. However, NSTIs of the head and neck usually have a monomicrobial profile and occur in mostly immunocompetent patients with a history of recent trauma/operation [12]. Antibiotic coverage for S. pyogenes and S. aureus using 1^st^/2^nd^ generation Cephalosporin is recommended, with additional coverage for Methicillin-resistant Staphylococcus aureus (MRSA) using Vancomycin/Daptomycin/Linezolid [12]. Delayed administration of antibiotics in sepsis is associated with a linear increase in the risk of mortality for each hour of delay [16]. In this case, Linezolid was appropriately administered, given possible concern for MRSA. However, the patient’s condition rapidly deteriorated as the progression of the infection likely outpaced pharmacological efforts.

The mainstay of NSTI management is emergent surgical debridement of all the necrosed and infected tissue, especially if there is concurrent systemic sepsis or multiorgan failure [12]. Multiple surgical debridements may be required [12]. The timing and extent of the first debridement serves as the most important factor in lowering mortality rates, as a delay of 24 h is associated with a ninefold increase in mortality [12]. In our case, the patient underwent emergent surgery within 38 min of arrival at the facility. However, despite urgent surgical management, there was already extensive spread of the infection across the scalp and eyelids. The initial nonspecific signs that delayed recognition and definitive management likely also significantly limited the scope for clinical recovery.

## Conclusion

Our case highlights the need for early recognition and management of NSTIs involving the head and neck, even when hallmark features are absent. Variable and nonspecific initial presentation with minimal superficial evidence, especially in the head and neck region, make early diagnosis and surgical evaluation challenging. However, it is important for surgeons to maintain a high clinical suspicion for NSTIs in patients with soft tissue infections of the head and neck and signs of systemic involvement, prompting immediate medical and surgical evaluation. Timeliness of intervention remains the single most important determinant of survival [12].
